# Honokiol, an activator of Sirtuin-3 (SIRT3) preserves mitochondria and protects the heart from doxorubicin-induced cardiomyopathy in mice

**DOI:** 10.18632/oncotarget.16133

**Published:** 2017-03-11

**Authors:** Vinodkumar B. Pillai, Abhinav Kanwal, Yong Hu Fang, Willard W. Sharp, Sadhana Samant, Jack Arbiser, Mahesh P. Gupta

**Affiliations:** ^1^ Department of Surgery, Pritzker School of Medicine, University of Chicago, Chicago, IL, USA; ^2^ Department of Medicine, Pritzker School of Medicine, University of Chicago, Chicago, IL, USA; ^3^ Department of Dermatology, Atlanta Veterans Administration Health Center, Emory University School of Medicine, Atlanta, GA, USA

**Keywords:** doxorubicin, cardiac toxicity, SIRT3, Cardiac hypertrophy, cancer therapy, Pathology Section

## Abstract

Doxorubicin is the chemotherapeutic drug of choice for a wide variety of cancers, and cardiotoxicity is one of the major side effects of doxorubicin treatment. One of the main cellular targets of doxorubicin in the heart is mitochondria. Mitochondrial sirtuin, SIRT3 has been shown to protect against doxorubicin-induced cardiotoxicity. We have recently identified honokiol (HKL) as an activator of SIRT3, which protects the heart from developing pressure overload hypertrophy. Here, we show that HKL-mediated activation of SIRT3 also protects the heart from doxorubicin-induced cardiac damage without compromising the tumor killing potential of doxorubicin. Doxorubicin-induced cardiotoxicity is associated with increased ROS production and consequent fragmentation of mitochondria and cell death. HKL-mediated activation of SIRT3 prevented Doxorubicin induced ROS production, mitochondrial damage and cell death in rat neonatal cardiomyocytes. HKL also promoted mitochondrial fusion. We also show that treatment with HKL blocked doxorubicin-induced cardiac toxicity in mice. This was associated with reduced mitochondrial DNA damage and improved mitochondrial function. Furthermore, treatments of mice, bearing prostrate tumor-xenografts, with HKL and doxorubicin showed inhibition of tumor growth with significantly reduced cardiac toxicity. Our results suggest that HKL-mediated activation of SIRT3 protects the heart from doxorubicin-induced cardiotoxicity and represents a potentially novel adjunct for chemotherapy treatments.

## INTRODUCTION

Honokiol (HKL) is a small molecular weight polyphenol derived from the tree, magnolia [1]. HKL's anti-cancer activity has been studied extensively [2]. HKL was found be effective in several animal models of cancer including lung, prostate, breast, colon and pancreatic cancers [3–7]. Besides its cytotoxicity in cancer, HKL was reported to induce variety of cytoprotective activities, including anti-inflammatory, anti-thrombotic, anti-arrhythmic, neuroprotective, anti-oxidative, and anti-hypertrophic effects [1, 8–10]. Thus, HKL has the potential to act both as an antitumor and cytoprotective molecule. Several molecular targets of HKL have been identified, these include NF-kb, STAT3, epidermal growth factor receptor, m-TOR, beta catenin and HIF1α [2]. Activities of all these enzymes are crucial for cell survival, proliferation and metabolism. Coincidently, SIRT3 also has a profound effect on regulating activity of these enzymes leading to the discovery that HKL is also an activator of SIRT3 [11–13].

SIRT3 is a NAD-dependent deacetylase localized primarily in mitochondria, and the only deacetylase in mitochondria that has robust deacetylase activity. A mass spectroscopic study revealed that nearly 65% of mitochondrial proteins were acetylated implying a crucial role of Sirt3 in mitochondria [14]. SIRT3 maintains mitochondrial health by deacetylating a wide variety of enzymes involved in metabolism, ROS production, apoptosis and mitochondrial dynamics [15]. In the heart, mitochondrial protein lysine hyper-acetylation was found to be associated with induction of heart failure, and overexpression of SIRT3 protected hearts against hypertrophic stimuli [16]. Consistent with this, Sirt3. KO mice develop cardiac hypertrophy spontaneously [17]. SIRT3 has been shown to augment Foxo3a mediated anti-oxidant defense mechanisms [17]. Several other mechanisms are also attributed to SIRT3-mediated cardioprotection. SIRT3 suppresses the activity of cyclophilin D, thereby blocking mitochondrial permeability transition pore opening [18]. SIRT3 can also block cardiac hypertrophy by augmenting the activity of LKB1, an upstream kinase of AMPK [13]. Furthermore, lipid accumulation-induced cardiac hypertrophy was mitigated by SIRT3 mediated deacetylation of long chain acyl CoA dehydrogenase (LCAD) [19].

SIRT3 also acts as a tumor suppressor [20]. Loss of SIRT3 results in increased ROS production, resulting in HIF1α stabilization, followed by transcriptional induction of various cancer promoting genes and a shift in metabolism towards glycolysis (Warburg effect). Mouse embryonic fibroblasts (MEF) overexpressing Myc/Ras developed into tumors when implanted in the hind limb of mice, whereas mice implanted with SIRT3^+/+^ Myc/Ras, SIRT3^−/−^ Myc, or SIRT3^−/−^ Ras MEFs did not, thus asserting tumor suppressor activity of SIRT3 [11]. The tumor suppressor role of SIRT3 in breast, colon, osteosarcoma and prostate cancer cells have been also reported [21]. Correspondingly, a recent study reported that HKL treatment by increasing the expression of SIRT3, attenuated palmitate induced decrease in SDH activity in LX2 cells [22]. These cells also showed reduced GPR91 and alpha SMA protein expression, markers of the fibrogenic response [22]. Similarly, we have recently reported that HKL treatment increases the activity of SIRT3 [10]. HKL not only blocked the pressure overload-mediated cardiac hypertrophic response, but also ameliorated pre-existing cardiac hypertrophy in mice. Additionally, HKL-treatment blocked cardiac fibroblast proliferation and differentiation into myofibroblasts in a SIRT3-dependent manner [10]. These findings suggested that whole body SIRT3 activation might concurrently reduce tumor progression while preventing the cardiac hypertrophic response.

Suppression of tumor growth, without damaging the heart, is an enigma researchers have been facing since the introduction of chemotherapy. Doxorubicin (Doxo) is a chemotherapeutic drug of choice for a wide variety of cancer types [23]. Optimal use of doxorubicin is limited by its cardiotoxicity [24]. Several mechanisms have been postulated to explain the cardiotoxic effects of doxorubicin. Even though oxidative stress is established as a primary cause of doxorubicin toxicity, interventions targeted to reduce oxidative stress have been found to be ineffective [25, 26]. Several oxidative stress independent mechanisms have also been proposed for doxorubicin-induced cardiotoxicity. In hearts, doxorubicin can form a ternary complex with topoisomerase II (Top2b) beta and DNA, inducing DNA double strand break and resultant cell death. Correspondingly, cardiomyocyte-specific deletion of Top2b has been shown to protect mice from doxorubicin-induced cardiotoxicity [27]. Another mechanism widely implicated in doxorubicin toxicity is the interaction of doxorubicin with iron, which generates substantial amount of ROS by several different mechanisms. Accordingly, overexpression of ABCB8, a protein that facilitates iron export from mitochondria, was shown to protect the heart from doxorubicin-induced cardiomyopathy in mice [28].

Apart from its acute toxicity, doxorubicin-induced cardiomyopathy may develop after years of latency, and these hypotheses are inadequate in explaining the etiology of chronic doxorubicin toxicity. Recently, our group and others have shown that activation of SIRT3 can protect the heart from doxorubicin-induced toxicity [29–31]. In cardiomyocytes doxorubicin preferentially accumulates in mitochondria and doxorubicin-induced cellular toxicity is associated with mitochondrial dysfunction; hence mitochondria-mediated mechanisms posit a potential etiology for the onset of delayed cardiotoxicity [28]. Childhood mitochondrial diseases are associated with progressive neurological, cardiac and liver dysfunction, whereas adult onset mitochondrial diseases manifest as a progressive multisystem disorders [32]. Mitochondrial DNA mutations are the primary cause for many mitochondrial diseases. Mitochondrial DNA is more vulnerable to damage and have more than 10-17 fold increased mutation rate than nuclear DNA [33]. One of the major DNA repair enzymes in mitochondria is OGG1. Decreased OGG1 expression in mitochondria is associated with mitochondrial dysfunction in hepatoma cells and tissues [34]. Cardiac overexpression of OGG1 protected mitochondrial DNA and reduced fibrosis following trans-aortic constriction, implying a critical role of OGG1 in blocking mitochondrial DNA damage [35]. Mitochondrial DNA damage is often associated with oxidation of guanosine nucleotide and accumulation of 8-oxo-dG adducts (7-8-dihydro-8-oxo-2 deoxyguanosine). OGG1 hydrolyses 8-oxo-dG adducts from the DNA and the presence of elevated 8-oxo-dG adducts in DNA represents deficiency in OGG1 activity [36]. Another mechanism that helps cells to maintain mitochondrial health and DNA integrity is mitochondrial fusion which enables the mixing of mitochondrial contents [37]. Content mixing enables the human cells to tolerate high levels of pathogenic mtDNA damage [38]. The major proteins involved in regulating the mitochondrial fusion-fission dynamics, includes DRP1 which promotes mitochondrial fission, whereas fusion events are regulated by two mitofusins, MFN1 and MFN2, which are responsible for fusion of outer mitochondrial membranes, and OPA1, which is involved in the fusion of inner mitochondrial membranes [37]. Recently, we found that over-expression of SIRT3 protects the heart from doxorubicin-induced toxicity while activating OPA1 to enhance mitochondrial fusion [31].

In this study, we report that HKL can protect cardiomyocytes from doxorubicin-induced cell death. We also demonstrate that HKL can block doxorubicin-induced cardiac hypertrophic response in mice without compromising the tumor killing potential of doxorubicin. Correspondingly, HKL attenuates pathologic cardiac hypertrophy and fibrosis by activating SIRT3.

## RESULTS

### Honokiol protects cardiomyocytes from doxorubicin-induced damage

**Figure 1:**
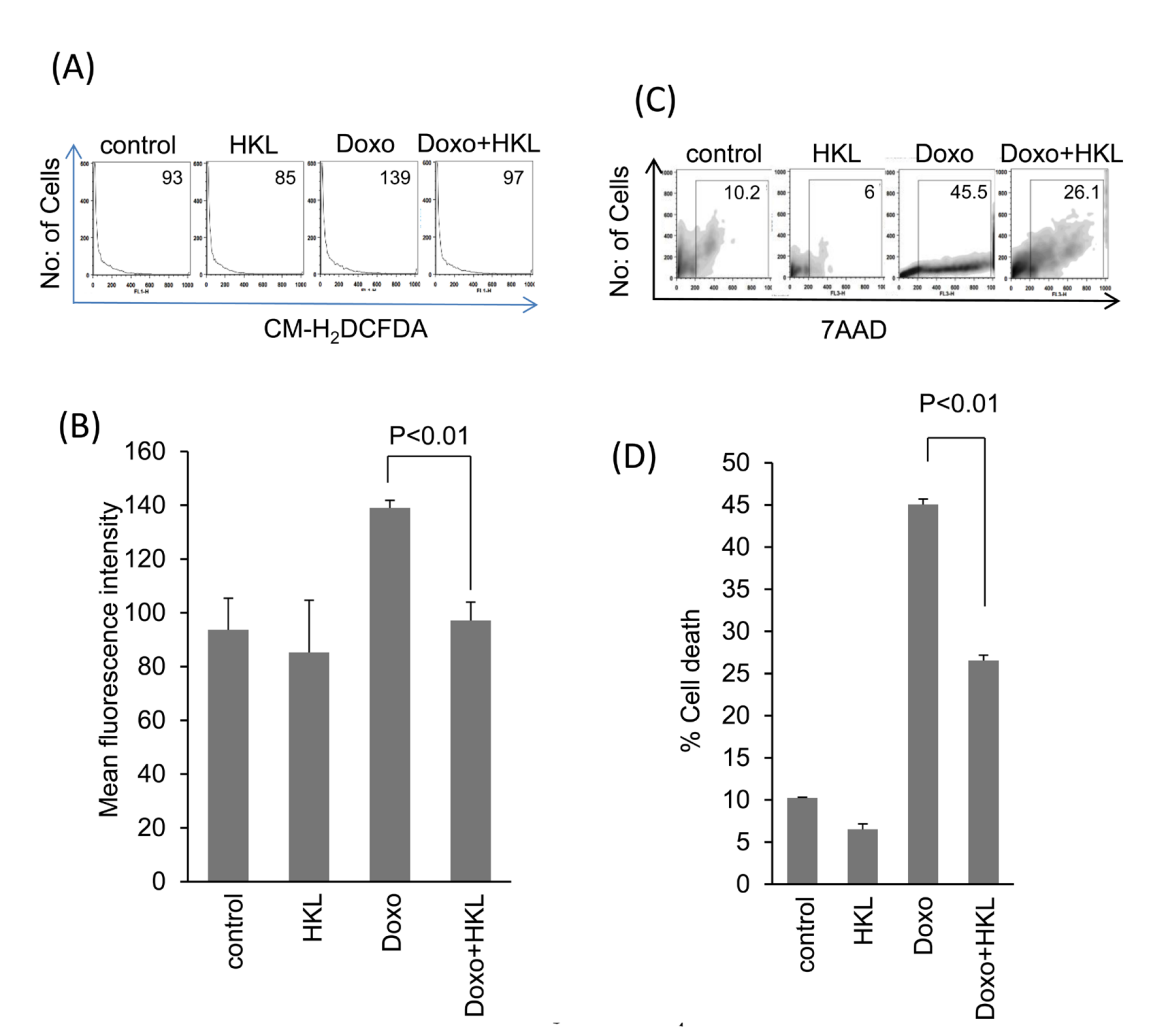
HKL treatment protects cardiomyocytes from doxorubicin-mediated injury **A**. Primary cultures of cardiomyocytes were treated with 10μM HKL for 24 hours in the presence or absence of 2μM doxorubicin. Cells were stained with CM-H_2_DCFDA and ROS levels were measured by fluorescence-activated cell sorter. **B**. Quantification of mean fluorescence intensity (MFI) in different groups of cells. Values are average of four independent experiments, Mean ± SE. **C**. Primary cultures of cardiomyocytes were treated with 10μM HKL for 24 hours in the presence or absence of 5uM doxorubicin. Extent of apoptosis was measured by estimating the percentage of 7AAD positive cells by FACS analysis. (D) Quantification of cell death in different groups of cells. Values are average of five independent experiments, Mean ± SE.

**Figure 2:**
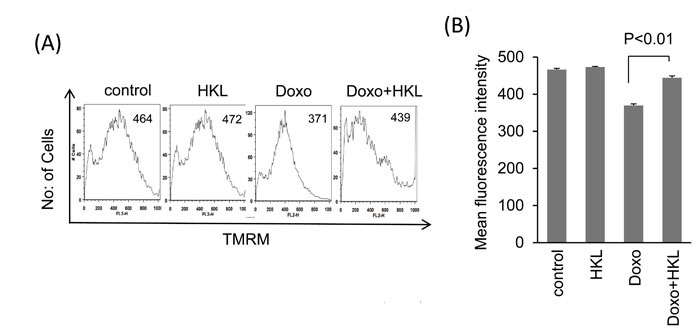
HKL preserves mitochondrial membrane potential **A**. Cardiomyocytes were treated with 2μM doxorubicin for 24-hours in the presence or absence of 10μM HKL. Cells were stained with TMRM and mitochondrial dye incorporation was measured by FACS analysis. **B**. Quantification of MFI of TMRM staining in different groups of cells. Values are average of three independent experiments, Mean ± SE.

We have previously shown that doxorubicin induces downregulation of SIRT3 and acetylation of mitochondrial proteins [29]. Treatment of cardiomyocytes with Doxo promotes ROS production, which can be alleviated by SIRT3 activation [29]. To test if HKL can effectively impede the Doxo-induced ROS production, cultures of cardiomyocytes were treated with Doxo in the presence or absence of HKL for 24 hours. Cells were stained with CM-H_2_DCFDA a non-fluorescent dye that fluoresces upon oxidation by ROS. We found that cells treated with HKL suppressed doxorubicin-induced ROS levels (Figure 1A and 1B). To support these findings, we performed the cell death experiments. Consistent with our ROS results, HKL treatment helped to rescue cardiomyocytes from doxorubicin-induced cell death (Figure 1C and 1D). To confirm these findings, we analyzed the mitochondrial membrane potential of doxorubicin treated cardiomyocytes in the absence or presence of HKL. TMRM is a cell permeant dye, which fluoresces when accumulated in the negatively charged polarized mitochondria of healthy cells. Doxorubicin treated cardiomyocytes showed reduced TMRM uptake, whereas pre-treatment with HKL rescued the TMRM uptake in doxorubicin treated cardiomyocytes, suggesting that HKL protects cardiomyocytes from Doxo-induced mitochondrial damage (Figure 2A and 2B).

**Figure 3:**
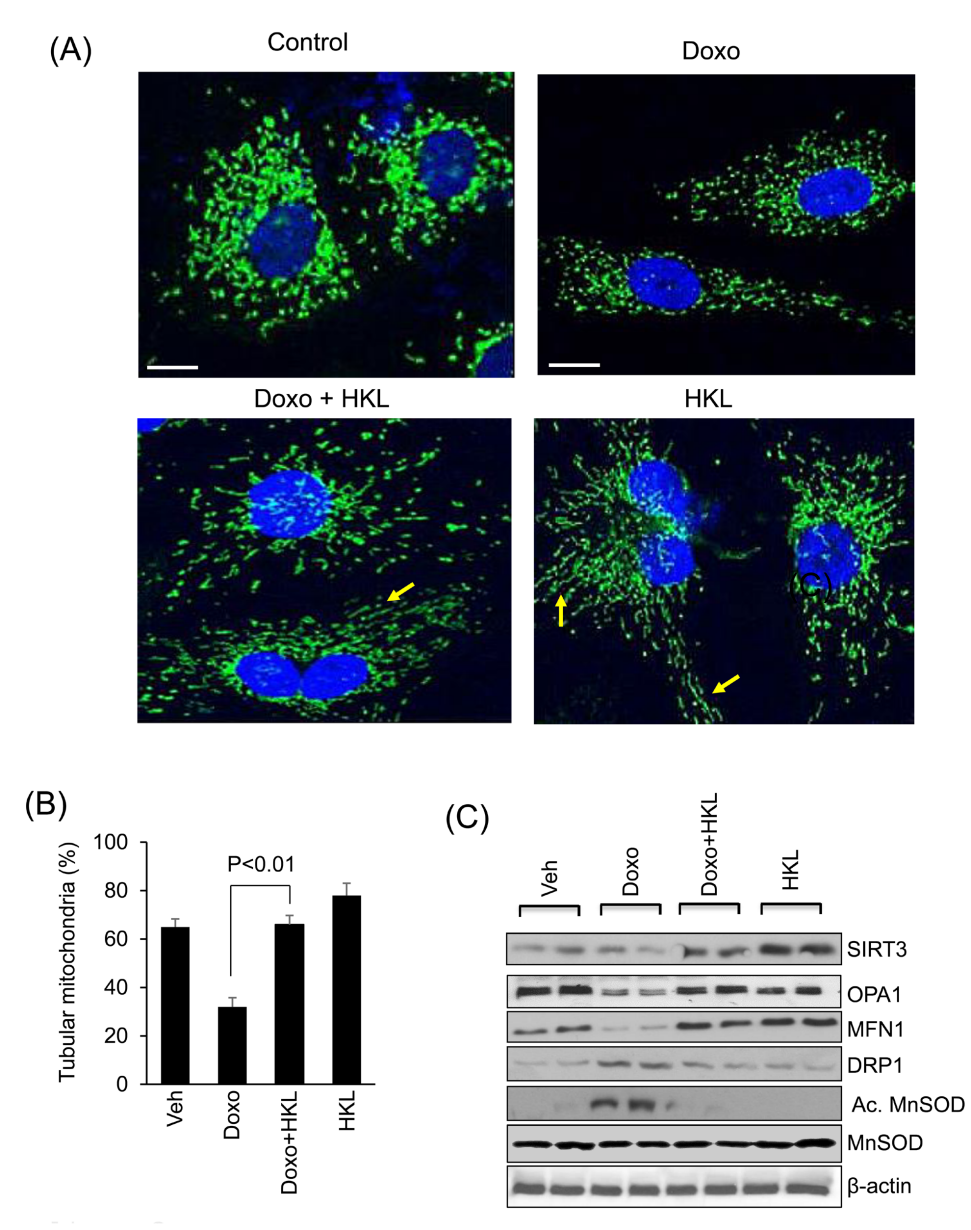
HKL promotes mitochondrial fusion **A**. Representative confocal images of cardiomyocytes treated with 5μM doxorubicin in the presence or absence of 10μM HKL. Mitochondria are visualized by overexpressing cells with mito-GFP adenovirus (green); scale bars, 10µm. **B**. Quantification of tubular mitochondria (arrows) in samples shown in Panel A. Values are average of three independent experiments Mean ± SE. **C**. Cell lysates of samples shown in panel A were prepared from another set of plates and subjected to immunoblotting with indicated antibodies. Representative blot of three independent experiments showing two different samples in each group (quantification of blots is given in supplement Figure 1).

Mitochondrial fusion/fission dynamics plays a critical role in maintaining mitochondrial health when cells are exposed to various stresses [39]. Mitochondrial fusion helps to alleviate stress by mixing contents of partially damaged mitochondria. SIRT3 activation promotes mitochondrial fusion, whereas, doxorubicin is known to induce mitochondrial fragmentation [31]. We therefore tested if HKL treatment can alleviate doxorubicin-induced toxicity by promoting mitochondrial fusion. Rat neonatal cardiomyocytes were overexpressed with Ad. Mito-GFP followed by treatment with 5µM doxorubicin for 24hr, and mitochondrial morphology was monitored by confocal microscopy. Doxorubicin treatment caused mitochondrial fragmentation, whereas treatment with HKL preserved the normal tubular shape of mitochondria, suggesting that HKL promotes mitochondrial fusion (Figure 3A and 3B). We also found reduced levels of MFN1 and OPA1, two fusion proteins involved in the fusion of outer and inner mitochondrial membranes, respectively. Pre-treatment with HKL helped to maintain SIRT3 as well as OPA and MFN-1 levels of cells exposed to doxorubicin. We also observed increased levels of mitochondrial fission protein, DRP1 in doxorubicin exposed cells, which was suppressed by HKL treatment (Figure 3C Supplementary Figure 1) [40]. SIRT3-mediated deacetylation at K-122 has been shown to increase enzymatic activity of MnSOD. Correspondingly, increased acetylation of MnSOD is correlated with loss of SIRT3 activity [41]. Consistent with this, we found increased activity of SIRT3 in Doxo plus HKL treated cells as revealed by decreased MnSOD acetylation (Figure 3C, Supplementary Figure 1). These results suggest that HKL mitigates mitochondrial damage by activating SIRT3 and promoting mitochondrial fusion.

### HKL protects mice from doxorubicin-induced cardiac hypertrophy

**Figure 4:**
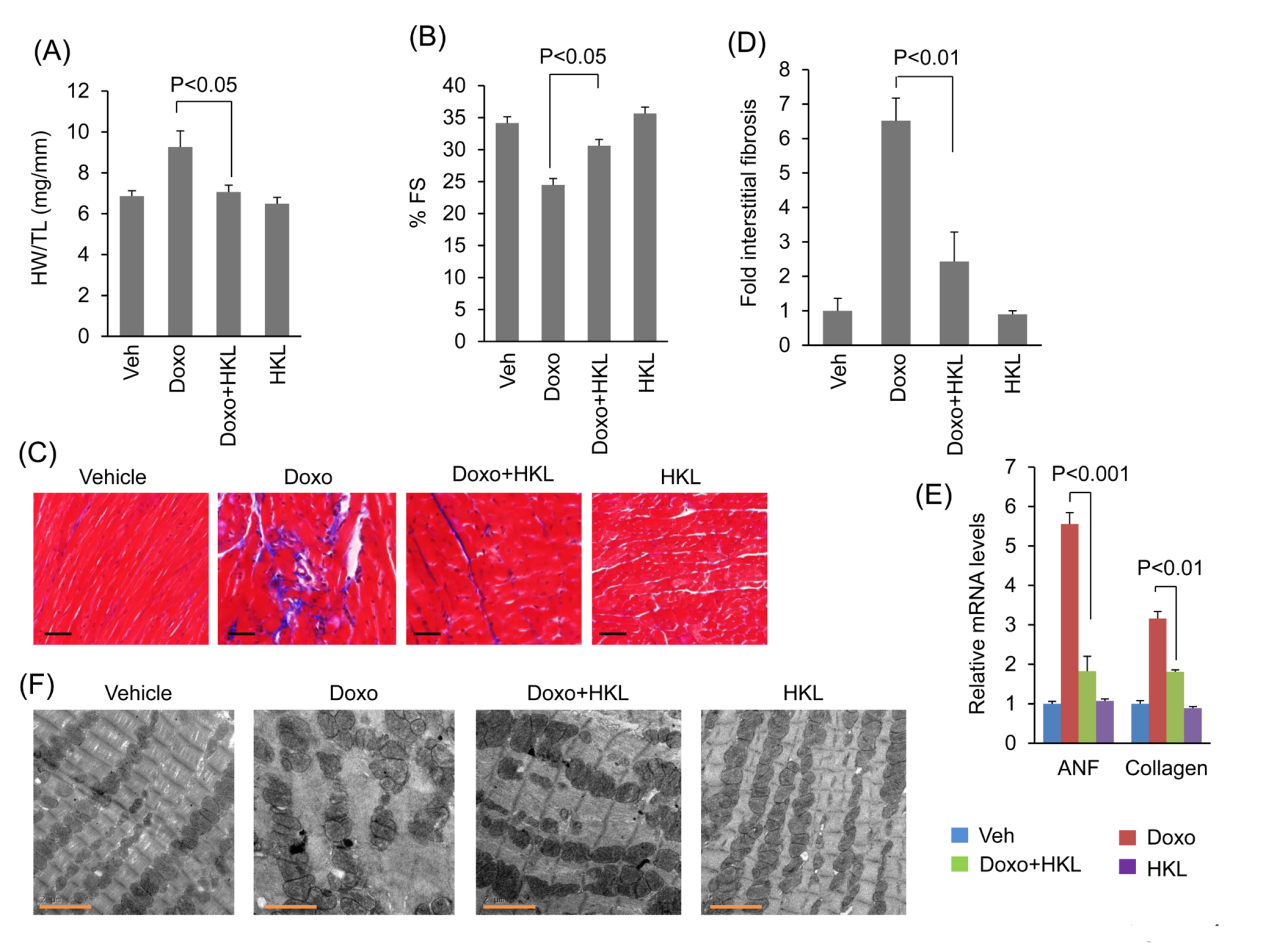
HKL treated mice are protected from doxorubicin-induced cardiac damage **A**. Heart weight to tibia length (HW/TL) ratio of vehicle (Veh), Doxo, Doxo plus HKL and HKL alone treated mice. Values are mean ± SE, *n* = 8-10. **B**. Echocardiographic measurements of fractional shortening in Veh, Doxo, Doxo plus HKL and HKL alone group of mice. Values are mean ± SE, *n* = 8-10. **C**. Representative sections of hearts stained with Masson's trichrome to detect fibrosis (*blue*); scale bars, 20 µm. **D**. Quantification of cardiac fibrosis in different groups of mice. Mean ± SE, *n* = 5. **E**. Expression levels of collagen-1 and ANF mRNA levels in different groups of mice, mean ± SE, n=6 mice. **F**. Representative electron microscopy images of the heart from Veh, Doxo, Doxo plus HKL and HKL alone treated mice. Scale bar 2µM.

**Figure 5:**
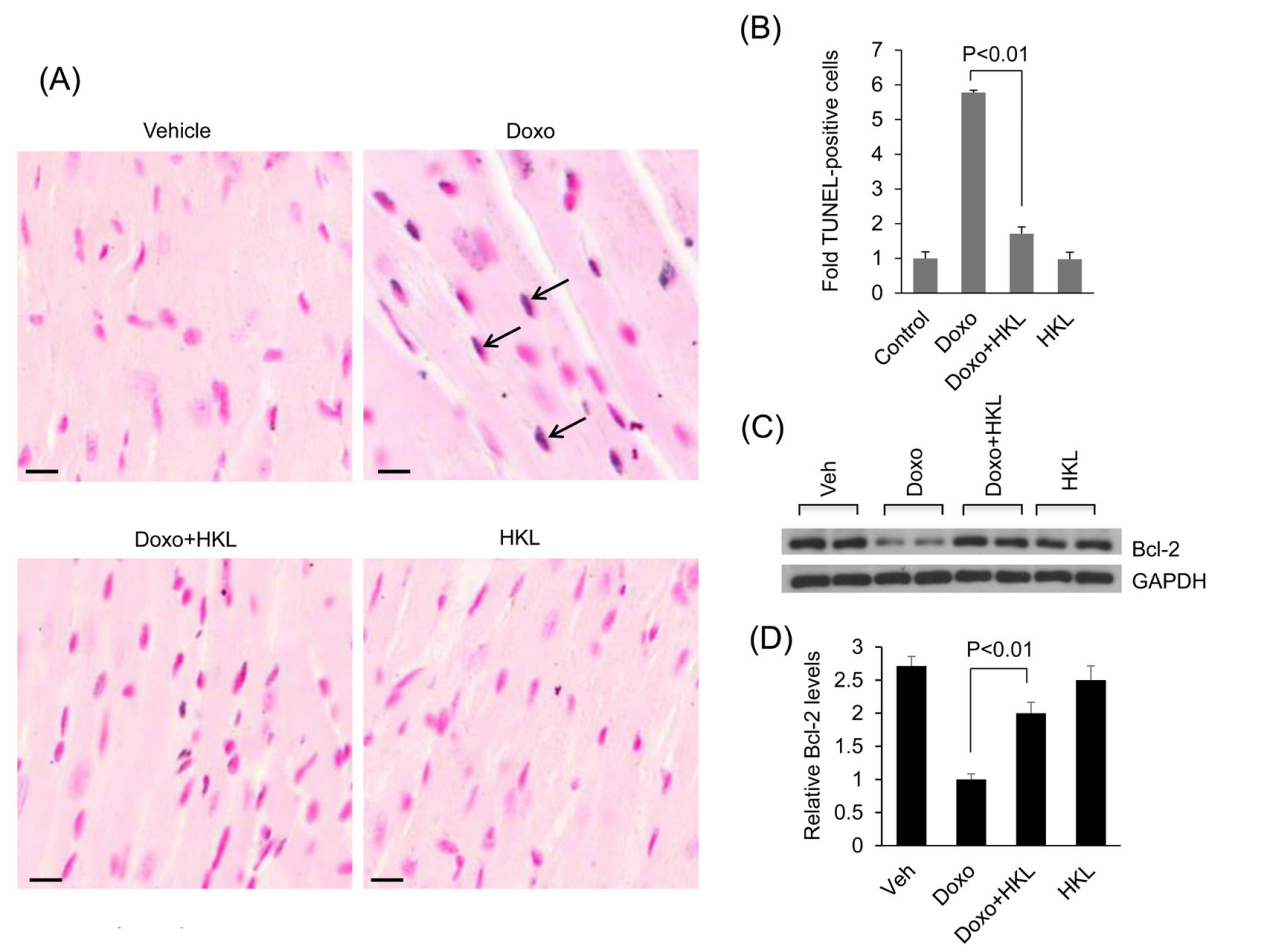
HKL treatment reduces doxorubicin induced cardiomyocyte apoptosis in mice **A.** Cardiomyocyte apoptosis was detected using TUNEL assay in different groups of mice. Arrows indicate TUNEL-positive cells: Scale bar 10μm. **B**. Quantification of apoptosis in mouse hearts. Mean ± SE, *n* = 5. **C**. Total heart lysate from different group of mice was analyzed by western blotting using anti-Bcl-2 antibody. Representative blot showing results of two mice in each group, *n* = 6. **D**. Quantification of relative Bcl-2 levels, Mean ± SE, *n* = 6.

Our *in vitro* data showed that HKL has cardio-protective effects against doxorubicin toxicity. To test this *in vivo*, mice were treated with three doses of doxorubicin (5mg/kg) at fifteen-day interval, and the control mice received vehicle injection. In the treatment group, HKL administration (0.2mg/kg/day) was started the day before doxorubicin infusion, and was maintained throughout the course of the study. In a previous study, this dose of HKL was found sufficient to protect mice from developing the TAC induced cardiac hypertrophic response [10]. Cardiac functions of mice were assessed 15 days after the last dose of Doxo. Doxorubicin injection resulted in 25% increase in heart weight/tibia length (HW/TL) ratio, whereas HKL treated mice showed no noticeable increase as compared to control (Figure 4A). Additionally, mice that received doxorubicin plus HKL showed preserved cardiac function, compared to doxorubicin and vehicle treated mice (Figure 4B). Consistent with this, doxorubicin plus HKL treated mice also showed significantly reduced fibrosis and reduced activation of fetal gene program as compared to doxorubicin alone treated group (Figure 4C, 4D and 4E). Electron microscopic examination of the heart sections revealed increased mitochondrial derangement and loss of myofibrils in doxorubicin treated group, as compared to the doxorubicin plus HKL treated group (Figure 4F). Cardiomyocytes death due to apoptosis and necrosis are considered as one of the main factors leading to doxorubicin-induced cardiotoxicity [42]. Hence we measured the apoptosis of myocytes using in situ terminal dUTP nick end labeling (TUNEL) assay. Doxorubicin treated mice showed significantly increased TUNEL positive cells, compared to mice receiving HKL together with Doxo (Figure 5A, 5B), suggesting that similar to our *in vitro* results, HKL was capable to block cardiomyocyte apoptosis *in vivo*. Bcl-2 is a mitochondrion associated anti-apoptotic protein, which helps to sequester pro-apoptotic proteins to inhibit their function [43]. We therefore examined the expression of Bcl-2 in heart lysates of different treatment groups of mice. In accordance with TUNEL assay, Bcl-2 levels were markedly reduced in mice treated with doxorubicin, compared to mice treated with Doxo together with HKL (Figure 5C, 5D), suggesting that HKL was capable to block doxorubicin-induced cardiomyocyte apoptosis in mice.

### HKL protects cardiomyocytes from doxorubicin-induced mitochondrial damage

**Figure 6:**
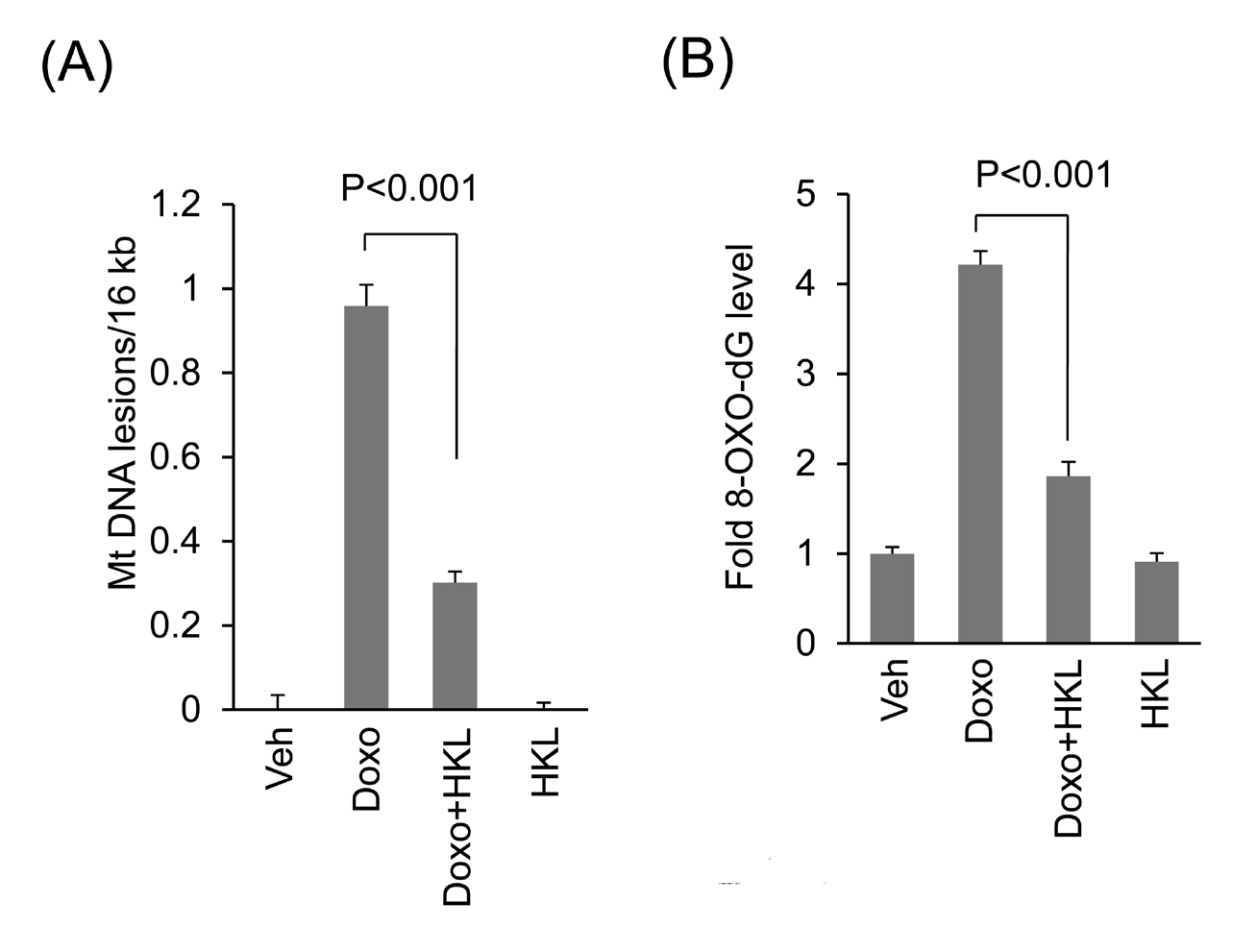
HKL treatment protects cardiomyocytes from doxorubicin-induced mitochondrial damage *in vitro* **A**. Primary cultures of cardiomyocytes were treated with 2μM doxorubicin in the presence or absence of 10μM HKL for 24 hours. mtDNA damage was assessed by quantitative PCR analysis. Mean ± SE, values are average of three independent experiments. **B**. Primary cultures of cardiomyocytes were treated with 10μM HKL for 24 hours in the presence or absence of 2μM doxorubicin and 8-oxo-dG content in total DNA was measured. Mean ± SE, values are average of three independent experiments.

Previous studies have also shown that SIRT3 protects mitochondria from doxorubicin-induced mtDNA damage [10, 22, 29, 44]. We therefore hypothesized that HKL might also protect mitochondrial DNA from doxorubicin induced damage. Intact mitochondrial DNA has the ability to support PCR amplification, whereas damaged DNA strand results in non-amplification of DNA. By utilizing this principle, we tested the effect of HKL on doxorubicin induced mtDNA damage in cardiomyocytes, treated with 2µM doxorubicin with or without 10µM HKL. HKL treatment significantly reduced Doxo-induced mitochondrial DNA lesions (Figure 6A). Increased DNA damage is also associated with increased levels of 8-oxo-dG adducts. To gain further evidence for this, we assessed the 8-oxo-dG levels in the DNA of doxorubicin or doxorubicin plus HKL treated cells. We observed reduced 8-oxo-dG levels in doxorubicin plus HKL treated cells, compared to doxorubicin alone treated cells, suggesting that HKL can protect cardiomyocytes from doxorubicin-induced mitochondrial DNA damage (Figure 6B).

**Figure 7:**
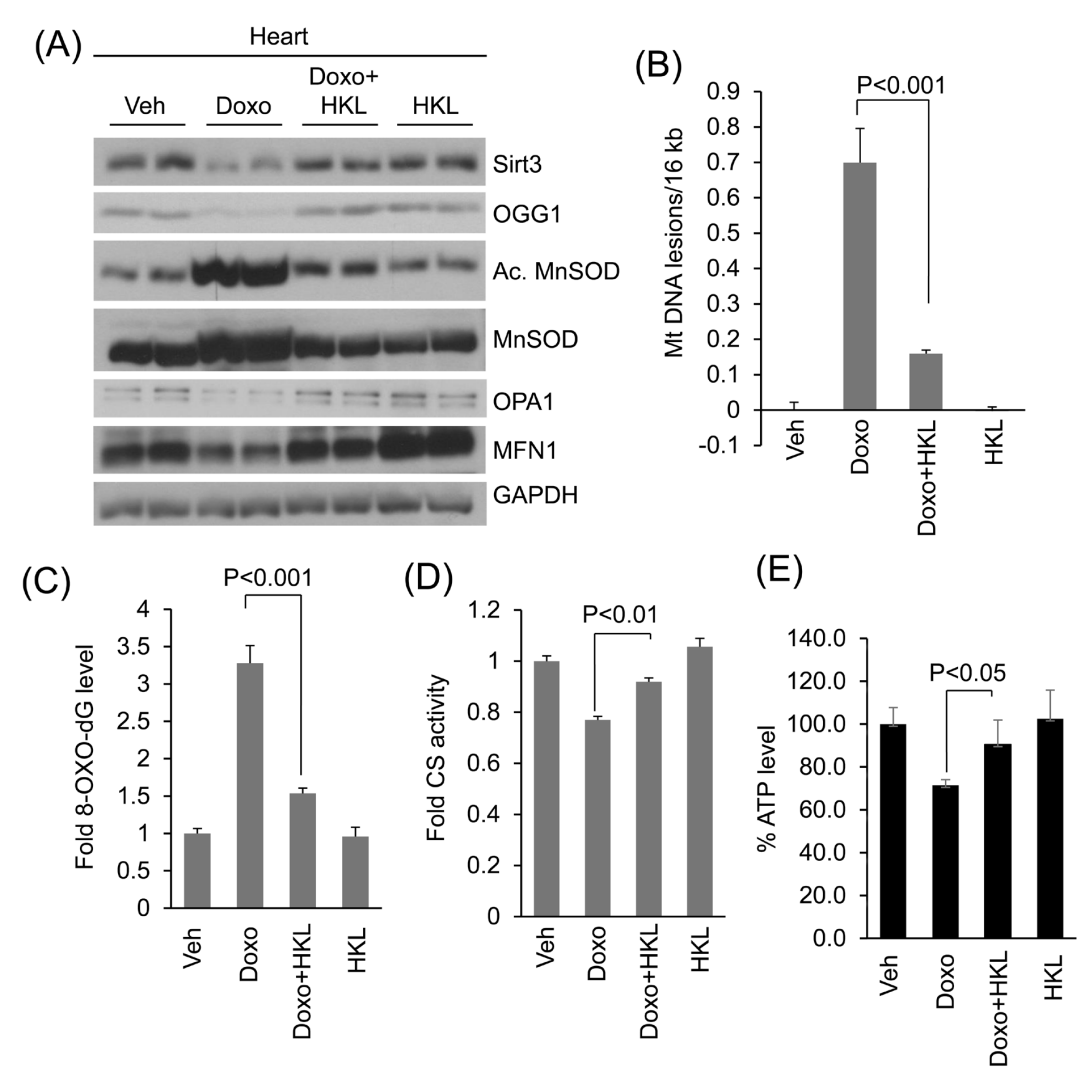
HKL treatment protects the heart from doxorubicin-induced mitochondrial damage *in vivo* **A**. Heart lysates of Vehicle, Doxo, Doxo plus HKL and HKL alone treated mice were subjected to immunoblotting using indicated antibodies. Representative blot of two different mice in each group are shown, *n* = 6. (quantification of blots is given in supplement Figure 2). **B**. Mitochondrial DNA damage was assessed in the whole heart of different group of mice as in panel A. All values are mean ± SE, *n* = 6. **C**. 8-Oxo-dG content in the DNA of whole heart of different group of mice. All values are mean ± SE, *n* = 5. **D**. Mitochondrial citrate synthase activity in the heart of different group of mice. CS, citrate synthase. Values are mean ± SE, *n* = 5. **E**. quantification of ATP contents in the heart lysate of different groups of mice as in *panel A*. Values are mean ± SE, *n* = 5.

We have previously shown that doxorubicin downregulates SIRT3 in mouse hearts with concomitant reduction in OGG1 levels [29]. Here, we tested if HKL treatment helps to maintain SIRT3 levels and activity in doxorubicin treated mice hearts (Figure 7A, Supplementary Figure 2). Consistent with our previous findings, we found reduced SIRT3 and OGG1 levels in doxorubicin treated mice hearts, whereas treatment with HKL helped to maintain both SIRT3 and OGG1 levels. We also found increased MnSOD acetylation in doxorubicin treated mouse hearts, which was again blocked in hearts treated with HKL, suggesting that HKL helped to maintain the SIRT3 activity following doxorubicin treatment (Figure 7A, Supplementary Figure 2). Consistent with the *in vitro* results, we also found increased expression of MFN1 and OPA1 in Doxo plus HKL treated hearts, compared to doxorubicin alone treated mice, thus showing the evidence for the ability of HKL to maintain the mitochondrial fusion dynamics (Figure 7A, Supplementary Figure 2). In agreement with this, we found that mice infused with doxorubicin, but treated with HKL showed reduced mitochondrial DNA damage and reduced accumulation of 8-oxo-dG, compared to mice infused with doxorubicin alone, suggesting that HKL has the potential to avert doxorubicin-induced mitochondrial DNA damage in mice (Figure 7B and 7C). Citrate synthase is a mitochondrial enzyme involved in the first step of TCA cycle. It catalyzes the condensation of acetate and oxaloacetate to form citrate, and hence a key marker for mitochondrial function. Doxorubicin treatment caused significant reduction in citrate synthase activity, whereas HKL treatment improved the activity of enzyme in doxorubicin treated mice (Figure 7D). We also measured ATP levels in these hearts, and found that, ATP levels were reduced by nearly 30% in Doxo-infused hearts, whereas they were generally maintained at control levels in HKL treated hearts (Figure 7E). Together, these results suggest that HKL mitigates Doxo-induced mtDNA damage and improves the mitochondrial function.

### HKL protected mouse hearts without affecting anti-cancer activity of doxorubicin

**Figure 8:**
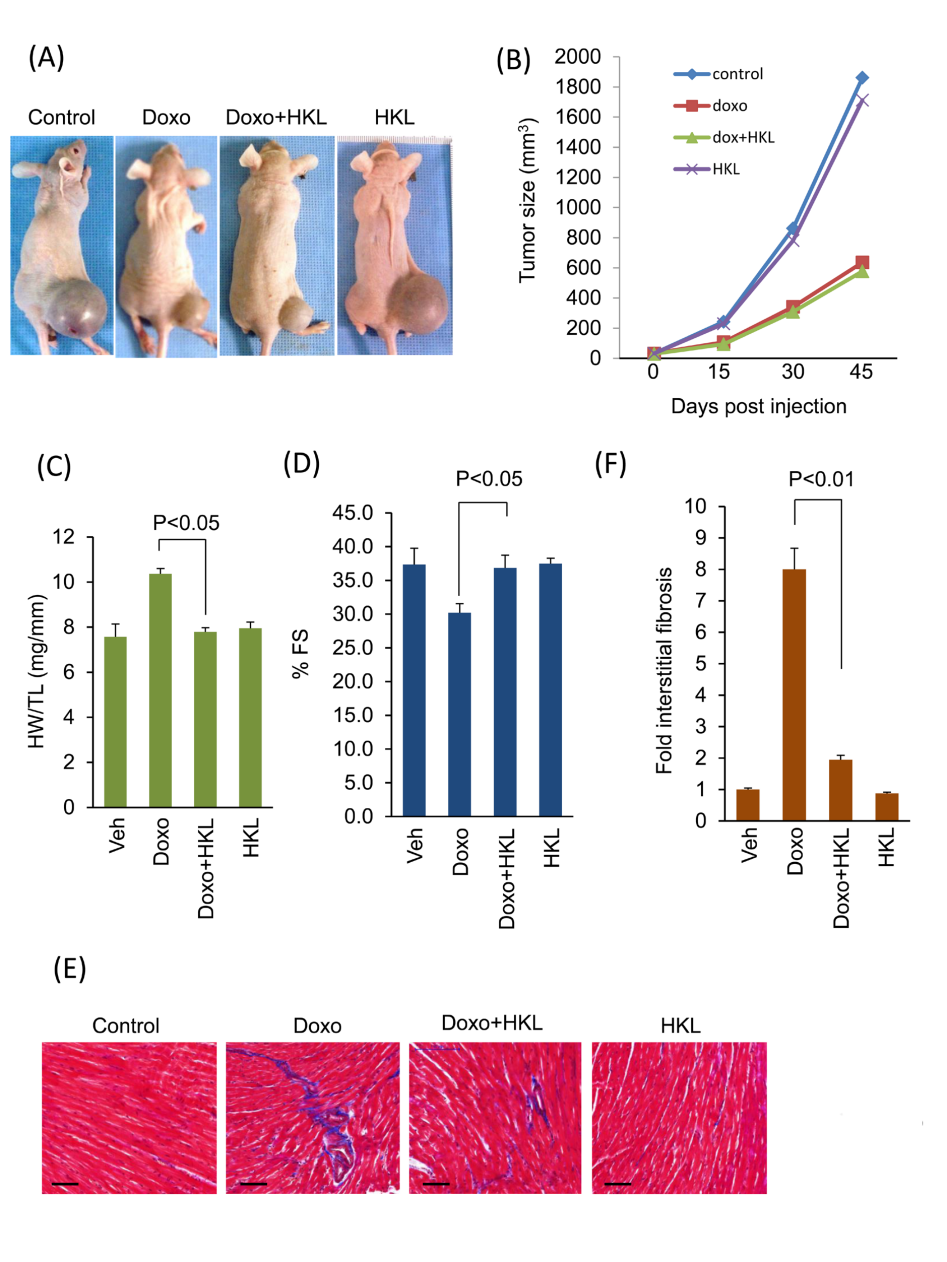
HKL treatment protected mouse hearts without affecting the anti-cancer potency of doxorubicin **A**. Representative images of mice implanted with PC3 cells and subjected to treatment with vehicle (control), Doxo, Doxo plus HKL and HKL alone. **B**. Tumor growth rate in mice of different treatment groups. **C**. Development of hypertrophy as measured by heart weight to tibia length (HW/TL) ratio in different treatments group of mice. values are mean ± SE, *n* = 8-10. **D**. Echocardiographic measurements of fractional shortening in mice. Values are mean ± SE, *n* = 8-10. **E**. Representative heart sections stained with Masson's trichrome to detect fibrosis (*blue*); scale bars, 20 µm.**F**. Quantification of cardiac fibrosis in different groups of mice as in panel E. Mean ± SE, *n* = 5.

Next, we sought to investigate whether treatment with HKL has any effect on the anti-tumor potency of doxorubicin. PC3 tumor cells were implanted subcutaneously in the flank of nude mice. Mice received first dose of doxorubicin seven days after tumor cell implantation. Two more doses of doxorubicin were given at 15-day intervals. HKL treatment was started one day before doxorubicin infusion. Mice treated with doxorubicin showed significant reduction in tumor volume as compared to controls. Similar reduction in tumor volume was observed in mice that were concomitantly treated with doxorubicin and HKL (Figure 8A and 8B). Similar to our earlier results, doxorubicin induced cardiac hypertrophy associated with reduced cardiac functions, but not when mice were treated with HKL during doxorubicin infusion (Figure 8C and 8D). Furthermore, doxorubicin plus HKL treatment also reduced cardiac fibrosis, suggesting that HKL can effectively block doxorubicin- induced cardiac hypertrophy without compromising the tumor killing potential of doxorubicin (Figure 8E and 8F).

## DISCUSSION

In this study, we report that HKL, an activator of SIRT3 can mitigate doxorubicin-induced cardiomyopathy in mice. Doxorubicin treatment downregulated SIRT3, leading to increased ROS levels and mitochondrial DNA damage. HKL mitigated Doxo-induced cardiac injury by activating SIRT3 and reducing cell death. This was associated with increased mitochondrial fusion and reduced mtDNA damage. We also found that HKL could protect the heart from doxorubicin toxicity without compromising the chemotherapeutic efficacy of the latter. Together, our studies reveal that HKL is a potent negative regulator of doxorubicin-induced cardiomyopathy.

Activation of sirtuins is known to protect the heart from doxorubicin-induced cytotoxicity [29–31, 45]. Among seven isoforms of the sirtuin family, SIRT3 is the major deacetylase present in mitochondria [46]. Sirt3-KO mice exhibit global hyper-acetylation of mitochondrial proteins, and accordingly, several disease models show deficiency of SIRT3. SIRT3 is downregulated in the muscles of diabetic mice, in the hearts of high fat diet fed mice as well as in the blood vessels of obese mice [47–49]. Moreover, in humans a SIRT3-polymorphism with loss of function is associated with pulmonary arterial hypertension and propensity to develop diabetes [50–52]. Consequently, reduced SIRT3 levels are reported in several models of cardiac hypertrophy and heart failure [10, 29]. Consistent with our previous results, we observed reduced SIRT3 levels in Doxo treated mouse hearts [29]. We have also previously shown that HKL physically binds to and augments the activity of SIRT3 [10]. In accordance with that, in the present study, we found that HKL treatment reversed the Doxo-induced hyper-acetylation of MnSOD.

SIRT3 deficiency is associated with increased ROS production which is detrimental to the cell [53]. Several factors could account for this effect of SIRT3. SIRT3 can directly deacetylate and activate MnSOD, an enzyme that is involved in the conversion of superoxide to hydrogen peroxide [41]. Further, SIRT3 can contain ROS levels by deacetylating and activating isocitrate dehydrogenase-2 (IDH2), an enzyme that consumes NADPH, thus providing NADPH to convert oxidized glutathione to glutathione, a major intracellular antioxidant [54]. In addition to this, SIRT3 negatively regulates ROS production by deacetylating and activating several components of the electron transport chain, which are believed to be responsible for 90% of the ROS production in mitochondria [55]. In accordance with these observations, we found that doxorubicin-induced cellular ROS was mitigated by the SIRT3 activator HKL.

Excessive ROS production is associated with oxidation of cellular macromolecules leading to cell death. SIRT3 can block cardiomyocyte apoptosis by activating many different defense mechanisms. Translocation of the protein Bax into the mitochondria causes apoptosis. Deacetylation of Ku70 by SIRT3 augments its interaction with the pro-apoptotic Bax and prevents its translocation to mitochondria [56]. Another process that influences apoptosis is mitochondrial dynamics. Constant mitochondrial fusion and fission permits the exchange of mitochondrial contents and Ca^2+^ signal transmission [39]. Hence, dysregulation of mitochondrial dynamics is detrimental to the cell. DRP1 is a master regulator of mitochondrial fission, and over activation of DRP1 is associated with mitochondrial fragmentation and cell death [57]. In contrast, MFN1 and OPA1 are proteins involved in outer and inner mitochondrial membrane fusion, respectively. SIRT3 mediated activation of OPA1 has been shown to protect cardiomyocyte from cell death [31]. In agreement with these findings, we found that doxorubicin treatment reduced protein levels of OPA1 and MFN1, while increasing the expression of DRP1. HKL treatment helped to maintain these protein levels at normal levels suggesting that HKL can improve the health of mitochondria by promoting mitochondrial fusion. Correspondingly, overexpression of SIRT3 also protects cardiomyocytes from Doxo-induced cell death [29]. Consistent with these observations, in this study, here we found that treatment with HKL efficiently blocks Doxo-induced cardiomyocyte cell death.

SIRT3 is a well-established anti-hypertrophic molecule. Deficiency of SIRT3 is associated with increased susceptibility to develop cardiac hypertrophy [17]. We have previously shown that Sirt3. Tg mice are resistant to develop agonist-induced as well as doxorubicin-mediated cardiac hypertrophy [17]. Further, HKL can block and reverse cardiac hypertrophic response in mice [10]. Consistent with these reports, in the present study we found that HKL could effectively block doxorubicin-induced cardiac hypertrophy and dysfunction. We have also shown previously that SIRT3 is a negative regulator of fibrosis, and Sirt3. KO mice have increased propensity to develop fibrosis in various organs including the heart with age [12]. HKL treatment has the potential to block proliferation of fibroblasts and their transition to myofibroblasts. Consistent with these observations we found reduced cardiac fibrosis in doxorubicin-induced mice treated with HKL. These results suggest that HKL can effectively block development of cardiac hypertrophy and fibrosis in response to the Doxo therapy.

**Figure 9:**
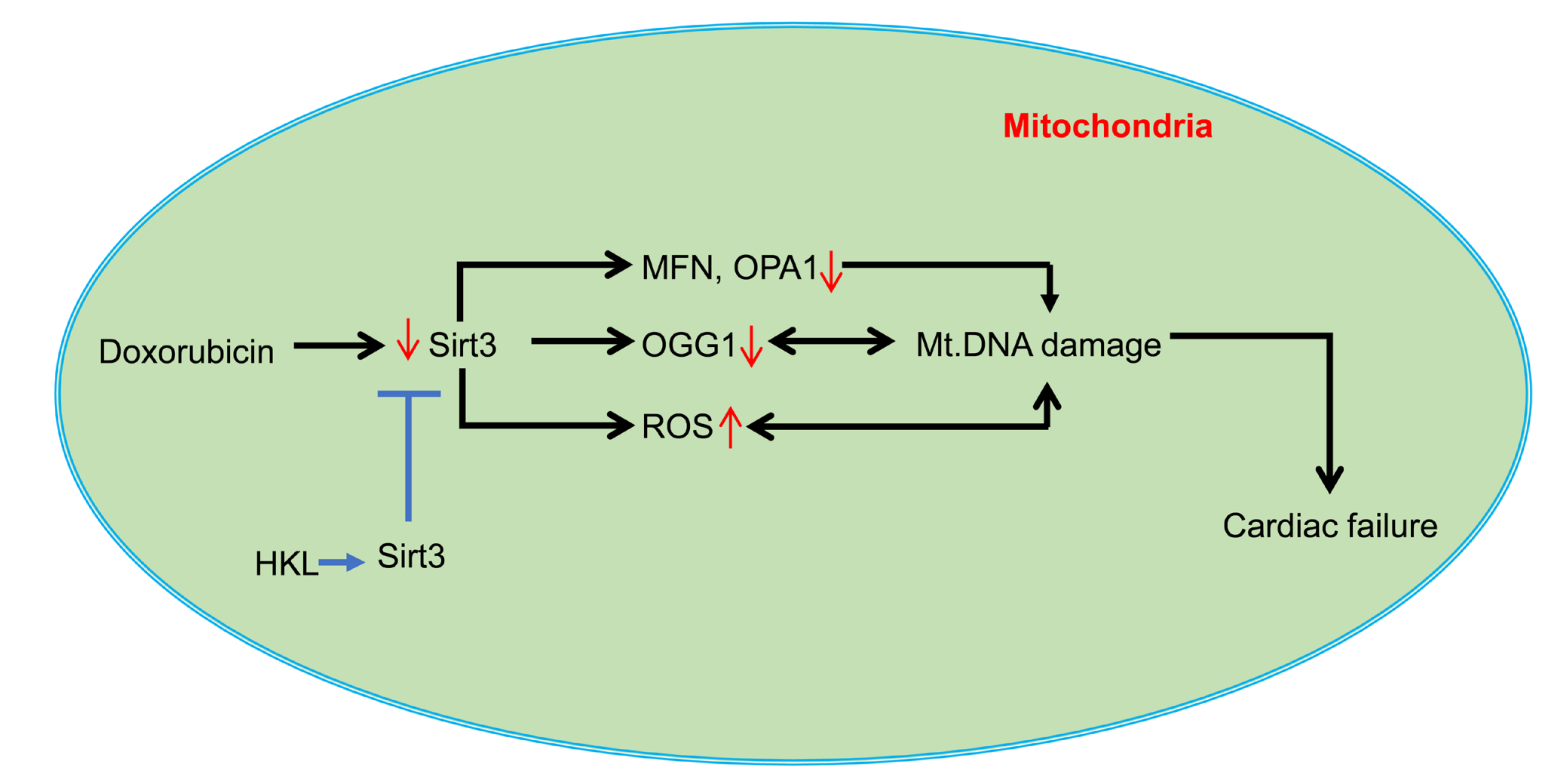
A model illustrating how HKL protects the heart from doxorubicin-induced cardiac injury: Doxorubicin-treatment downregulates SIRT3 in the heart resulting in decreased levels of OGG1, MFN1 and OPA1, and increased levels of ROS in mitochondria, all contributing to mtDNA damage Doxorubicin can also cause mtDNA damage by directly binding to DNA. Activation of Sirt3 by HKL augments OGG1, MFN1 and OPA1 and reduces ROS production, thereby mitigating mtDNA damage by decreasing the oxidative damage as well by increasing the efficiency of DNA repair and mitochondrial fusion dynamics. These changes promote overall health of mitochondria, and thereby protecting cardiac myocytes from death and development of cardiac failure.

Mitochondria contribute to more than 90% of the ROS generated in cells having high respiratory rates like cardiomyocytes [58]. Mitochondrial DNA, because of its proximity to the source of ROS is highly vulnerable to damage. It is also reported that damages to mitochondrial DNA are more extensive and persist longer than nuclear DNA damage following oxidative stress [59]. Mitochondrial DNA also has a 10-17 fold higher mutation rate, compared to nuclear DNA [33]. Doxorubicin-induced mitochondrial superoxide production can cause damage to mitochondrial DNA [28, 60]. One of the most abundant products of DNA nucleotide damage is synthesis of 7-8-dihydro-8-oxo-2 deoxyguanosine (8-oxo-dG) [61]. We have previously shown that doxorubicin treatment is associated with increased mtDNA damage and accumulation of 8-oxo-dG [29]. Accumulation of 8-oxo-dG is correlated with reduced levels of OGG1, a mitochondrial DNA repair enzyme. Over expression of SIRT3 reduced mitochondrial DNA damage and maintained OGG1 levels [29, 62]. Similar to this observation in the current study, HKL treatment reduced mitochondrial DNA damage, decreased 8-oxo-dG levels and increased mitochondrial functions. Mitochondrial DNA mutations are directly associated with the etiology of many diseases. Co-existence of the disease causing mutant DNA with wild type DNA is defined as heteroplasmy. Cells can tolerate mtDNA mutations to a large extent, until the wild type to mutant DNA ratio increases above certain threshold for manifestation of the clinical symptoms. This varies with mutations and tissue types having a value in the range of 60-90% [63]. Mitochondrial DNA mutations increase exponentially in post-mitotic tissues [33]. Delayed onset of doxorubicin-induced cardiotoxicity could be because of the time required to reach the threshold ratio of mitochondrial DNA mutations needed for dysfunction of the heart. Further, because fission and fusion processes help to redistribute mitochondrial metabolites, enzymes, gene-products and DNA, it is rational to hypothesize that defects in mitochondrial dynamics may also contribute to perpetuation of mtDNA damage and development of delayed cardiotoxicity (Figure 9) [64]. Our findings suggests that HKL mediated mitochondrial protection may alleviate delayed cardiac toxicity. In this context, it is also imperative to consider that HKL has off target effects on many other critical proteins, including NF-kb, STAT3, mTOR, and EGFR, which could have added to the observed effects [2].

Apart from its role in cardiac protection, SIRT3's role in cancer metabolism is also well studied. SIRT3 is a critical regulator of Warburg effect, a shift in metabolism towards glycolysis in the presence of oxygen [11]. Sirt3. KO mouse embryonic fibroblasts showed increased glycolysis and decreased mitochondrial oxidative metabolism, and mice deficient in SIRT3 showed increased propensity to develop cancers [20]. SIRT3 acts as a tumor suppressor by inhibiting ROS production and activation of HIF1α. SIRT3 functions as a tumor suppressor in a variety of cancers including B cell malignancies, prostate cancer and hepatocellular carcinoma. Contrary to these observations, activation of SIRT3 can also confer protection to cancer cells. Increased expression of SIRT3 was found in oral cancer and melanoma [65, 66]. In the present study, we did not find significant difference in tumor volume between doxorubicin alone or doxorubicin and HKL treated mice, suggesting that cancer cell protective function of SIRT3 is not activated in HKL treated cells. HKL is also well documented for its role as an anticancer drug. HKL has been shown to reduce various types of tumors in different animal models. In the present study, we did not see any addictive effect of HKL on doxorubicin's cancer killing potential. This could be because of the very low dose of HKL used in our study (0.2mk/kg/day), compared to higher doses in other studies [67, 68].

In summary, our results suggest that doxorubicin-induced toxicity to the heart is associated with reduced SIRT3 levels. HKL, an activator of SIRT3 can mitigate Doxo-induced cardiotoxicity by maintaining SIRT3 levels, and attenuating Doxo-induced mtDNA damage. Based on our results we believe that HKL has the potential to protect the heart from Doxo-induced toxicity without compromising its chemotherapeutic potential.

## MATERIALS AND METHODS

### Cardiomyocyte culture

Primary cultures of cardiac myocytes were prepared from neonatal rat hearts. In brief, hearts were removed from 2- to 3-day-old pups (Sprague-Dawley rats, either sex) and kept in cold DMEM. Ventricles were cut into 4 to 6 evenly sized pieces using small scissors and digested using collagenase type II (Worthington). The digested solution was collected with the cannula-syringe, avoiding the tissue chunks and was added to one of the already aliquoted 10 ml FBS (100%). These steps were repeated six to seven times till no tissue chunks are visible. Tissue digest was spun and cell pellet was resuspended in DMEM with 5% FBS. Cells were pre-plated for 1 h to remove fibroblasts, and unattached cardiomyocytes in suspension were collected and plated in fibronectin-coated culture plates. Cardiomyocytes cultures were used after 24 h of plating.

### Purificarion of honokiol

Honokiol was purified and prepared as described previously [69].

### Reactive oxygen species (ROS) detection

ROS levels were detected using CM-H_2_DCFDA (Invitrogen) as per the manufacturer's instructions. Briefly, Primary cultures of cardiomyocytes were treated with 10 μM HKL for 24 hours in the presence or absence of 2 μM doxorubicin. Cells were stained with CM-H_2_DCFDA. Cells were acquired by FACSCalibur and analyzed with use of FlowJo. The mean fluorescence intensity of cells positive for CM-H_2_DCFDA staining was determined.

### Cell death assay

Cardiomyocytes were cultured in 6-well plates and were treated with 5 μM doxorubicin for 24 hours. Cells were harvested from tissue culture plates and centrifuged at 1,000 rpm for 5 min at 4°C. Supernatant was removed, and cells were washed twice with cold PBS. Cells were then resuspended in 100μl of cold 1x binding buffer, and 20μl of 7AAD (BD) was added. Samples were incubated for 15 min in the dark at room temperature. A total of 400 μl of 1× binding buffer was added and analyzed by flow cytometry using a FacScan analyzer (Becton-Dickinson, San Jose, CA). Results were processed using FlowJo software.

### TMRM uptake

To monitor mitochondrial membrane potential (ΔΨ_m_), tetramethyl rhodamine methyl ester (TMRM; Invitrogen), a ΔΨ_m_-dependent cationic dye, was used. In brief, primary cultures of cardiomyocytes were treated with 10 μM HKL for 24 hours in the presence or absence of 2 uM doxorubicin. Cells were stained with TMRM, acquired by FACSCalibur and analyzed with use of FlowJo. The mean fluorescence intensity of cells positive for TMRM staining was determined.

### Mitochondrial morphology

Rat cardiomyocytes were overexpressed with Ad. Mito-GFP (kindly provided by Paul Schumacker from Northwestern University, Evanston, IL) and mitochondrial morphology was determined as reported elsewhere [70, 71]. Briefly, 100 cells were randomly selected for each treatment group and were designated as being either elongated all over (100%), predominantly elongated (80%), modestly elongated (60%), predominantly fragmented (40%), or fragmented mostly (20%). Three independent experiments by two investigators blinded to the treatment were carried out. Confocal microscope was used to visualize the mitochondria.

### Citrate synthase activity assay

Citrate Synthase activity was measured using citrate synthase activity kit (BioVison Inc).

### Mitochondrial DNA damage assay

Genomic DNA was isolated using Qiagen Genomic-tip 20/G and Qiagen DNA Buffer Set (Qiagen, Gaithersburg, MD) per the manufacturer's instruction. Eluted DNA was incubated with isopropanol overnight at -80°C and centrifuged 12,000g for 60 min. DNA was washed with 70% ethanol and dissolved in TE buffer. PCR was performed using Ex-taq (Clonetech, Mountain View, CA). Primer sequences for long PCR are: forward, 5’ cccagctactaccatcattcaagtag3’ and reverse, 5'gagagattttatgggtgtaatgcggtg3’. Short PCR was performed using forward primer sequence 5`gcaaatccatattcatccttctcaac3` and the reverse primer sequences same as long PCR. Resultant PCR products were quantified using Pico-green (Life Technologies). Values obtained from the long fragments were normalized using values from short fragments. The lesion frequency per amplicon was then calculated as *λ* = −ln(*A*D/*A*O), where *A*D/*A*O is the ratio of amplification of the treated samples (*A*D) to the amplification of the control samples (*A*O).

### 8-Oxo-dG level estimation

8-Oxo-dG levels were estimated using 8-Oxo-dG ELISA kit (Trevigen) as per manufacturer's instructions.

### Antibodies and immunoblotting

The OGG1 antibody was purchased from Novus Biologicals. The actin, GAPDH and MFN1 antibodies were from Santa Cruz Biotechnology. MnSOD antibody was from Millipore. Ac-K^122^MnSOD antibody was generated in Dr David Gius lab (Northwestern University). Anti-SIRT3 and Anti-acetyl lysine antibody was from cell signaling. All other antibodies were purchased from BD biosciences. Cells or heart ventricular tissue lysates were prepared in the RIPA buffer [50 mM Tris·HCl (pH 7.5), 0.1% Nonidet P-40, 1% Triton X-100, 150 mM NaCl, 1 mM EDTA, 100 mM phenylmethylsulfonyl fluoride (PMSF), 5 mM sodium orthovanadate, 10 mM β-glycerol phosphate, and 20 mM NaF and Sigma protease inhibitors]. Typically, 20-50 μg of protein lysates was used for immunoblots.

### ATP estimation

ATP levels were estimated by using ATP determination Kit (Molecular Probes) according to manufacturer's instructions.

### Real-time PCR analysis for mRNA levels

Total RNA was isolated from mouse hearts by using Trizol Reagent (Invitrogen). The residual genomic DNA was digested by incubating the RNA preparation with 0.5 units of RNase-free DNase-1 per microgram of RNA in 1 × reaction buffer for 15 min at room temperature, followed by heat inactivation at 90°C for 5 min. Two micrograms of DNase-treated RNA were reverse transcribed by use of Fermentas, RevertAid First Strand cDNA Synthesis Kit. The resultant cDNA was diluted 10-fold before PCR amplification. A reverse transcriptase minus reaction served as a negative control. The mRNA levels were measured by SYBR green real-time PCR. Primer sequences, ANF forward 5'TCGTCTTGGCCTTTTGGCT3’ and reverse 5'TCCAGGTGGTCTAGCAGGTTCT3’. Collagen-1 Forward 5'AAACCCGAGGTATGCTTGATCTGTA3’ and reverse 5’ GTCCCTCGACTCCTACATCTTCTGA3’.

### TUNEL assay

TUNEL assay was performed on heart sections using CardioTACS In Situ Apoptosis Detection Kit (R&D Systems Inc, USA) according to the manufacturer's instructions.

### Doxorubicin treatment of mice

For *In vivo* tumor study, tumors were generated in Athymic male BALB/c mice from Envigo, USA by s.c. injection of PC-3 cells (1× 10^6^ cells) with 50-μL matrigel matrices (Corning). Seven days after tumor cell implantation, mice were given first dose of doxorubicin. Doxorubicin reconstituted in 0.85% sterile sodium chloride was administered by intraperitoneal injection. Control animals were also treated simultaneously with identical volume of 0.85% sterile NaCl. Mice were treated with 5mg/kg doxorubicin every 15 days for a total of three doses (Cumulative dose 15mg/kg body weight). 15 days after the last dose of doxorubicin cardiac hypertrophy and heart functions of mice were studied. HKL treatment (0.2mg/kg/day, IP) was started the day before doxorubicin infusion and was maintained throughout the course of the study. Tumor volume was calculated by ab^2^/2 where “a” and “b” are the long and short axes of the tumor. The Institutional Animal Care and Use Committee of the University of Chicago approved all the animal protocols.

### Measurement of mouse heart functions

Chest hair of mice were removed with a topical depilatory agent and transthoracic echocardiography was performed under inhaled isoflurane (~1%) for anesthesia, delivered via nose cone. Limb leads were attached for electrocardiogram gating, and the animals were imaged in the left lateral decubitus position with a VisualSonics Vevo 770 machine, using a 30 MHz high-frequency transducer. Body temperature was maintained using a heated imaging platform and warming lamps. Two-dimensional images were recorded in parasternal long- and short-axis projections, with guided M-mode recordings at the midventricular level in both views. LV (left ventricle) cavity size and wall thickness were measured in at least three beats from each projection and averaged. LV wall thickness (interventricular septum [IVS] and posterior wall [PW] thickness) and internal dimensions at diastole and systole (LVIDd and LVIDs, respectively) were measured. LV fractional shortening ([LVIDd-LVIDs]/LVIDd) and relative wall thickness ([IVS thickness+PW thickness]/LVIDd) were calculated from the M-mode measurements.

### Statistical analysis

Statistical differences among groups were determined with either Student's *t*-test (for two groups) or one-way analysis of variance (ANOVA). *P* values less than 0.05 were considered significant.

## SUPPLEMENTARY MATERIALS FIGURES


