# Supplementary material for: Honokiol, an activator of Sirtuin-3 (SIRT3) preserves mitochondria and protects the heart from doxorubicin-induced cardiomyopathy in mice
Source: Oncotarget. 2017 Mar 11;8(21):34082–98. doi: 10.18632/oncotarget.16133 (PMC5470953; doi:10.18632/oncotarget.16133)
Supplement: Supplementary file 1 [file oncotarget-08-34082-s001.pdf]

Honokiol, an activator of Sirtuin-3 (SIRT3) preserves mitochondria and protects the heart from doxorubicin-induced cardiomyopathy in mice

Supplementary Material

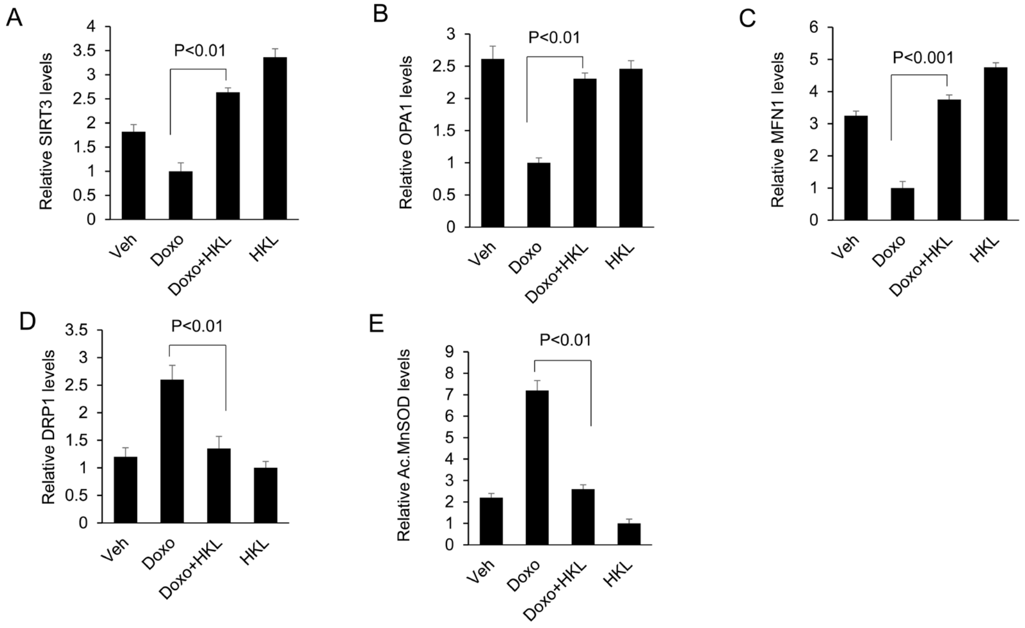

Quantification of relative SIRT3, OPA1, MFN1, DRP1 and acetylated (Ac) MnSOD levels in cardiomyocytes treated as indicated. Values are average of three independent experiments, mean±S.E.

Supplementary Figure 1

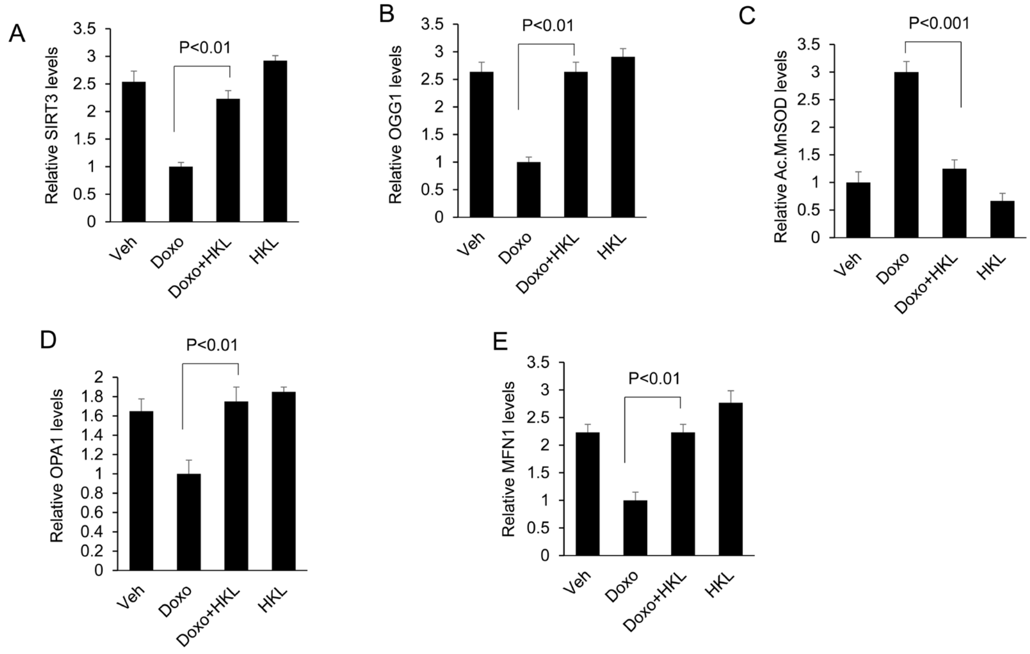

Quantification of relative SIRT3, OGG1, acetylated (Ac) MnSOD, OPA-1 and MFN-1 levels in mice treated as indicated., mean±S.E. n=6 mice

Supplementary Figure 2
